# Tailored Multivalent Targeting of Siglecs with Photosensitizing Liposome Nanocarriers

**DOI:** 10.1002/anie.202206900

**Published:** 2022-06-21

**Authors:** Verónica Almeida‐Marrero, Fleur Bethlehem, Sara Longo, M. Candelaria Bertolino, Tomás Torres, Jurriaan Huskens, Andrés de la Escosura

**Affiliations:** ^1^ Department of Organic Chemistry Universidad Autónoma de Madrid Campus de Cantoblanco 28049 Madrid Spain; ^2^ Department of Molecules & Materials MESA+ Institute for Nanotechnology Faculty of Science and Technology University of Twente P.O. Box 217 7500 AE Enschede The Netherlands; ^3^ Institute for Advanced Research in Chemistry (IAdChem) Campus de Cantoblanco 28049 Madrid Spain; ^4^ Tomás Torres IMDEA Nanoscience Campus de Cantoblanco 28049 Madrid Spain

**Keywords:** Liposome Nanocarriers, Multivalency, Photosensitizer, Siglecs

## Abstract

The modification of surfaces with multiple ligands allows the formation of platforms for the study of multivalency in diverse processes. Herein we use this approach for the implementation of a photosensitizer (PS)‐nanocarrier system that binds efficiently to siglec‐10, a member of the CD33 family of siglecs (sialic acid (SA)‐binding immunoglobulin‐like lectins). In particular, a zinc phthalocyanine derivative bearing three SA moieties (**PcSA**) has been incorporated in the membrane of small unilamellar vesicles (SUVs), retaining its photophysical properties upon insertion into the SUV's membrane. The interaction of these biohybrid systems with human siglec‐10‐displaying supported lipid bilayers (SLBs) has shown the occurrence of weakly multivalent, superselective interactions between vesicle and SLB. The SLB therefore acts as an excellent cell membrane mimic, while the binding with PS‐loaded SUVs shows the potential for targeting siglec‐expressing cells with photosensitizing nanocarriers.

## Graphical abstract

A promising approach towards optically active biohybrid systems that selectively bind receptors of the sialic acid‐binding immunoglobulin‐like lectins (siglecs) family is described. To this end, siglec‐modified supported lipid bilayers act as cell membrane mimics that interact in a multivalent fashion with photosensitizer‐loaded vesicles, showing a potential for targeting siglec‐expressing cells with photosensitizing nanocarriers.

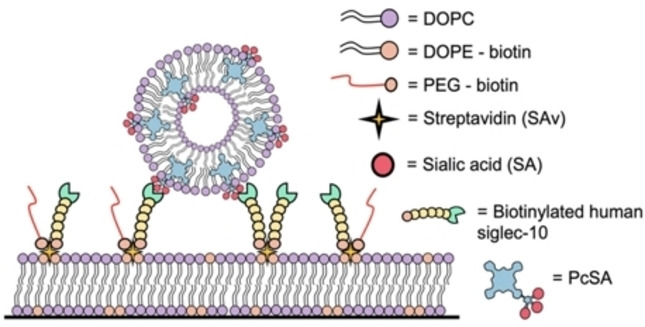

## Introduction

Liposomes, vesicles with an aqueous lumen formed by self‐assembly of a phospholipid bilayer, represent excellent nanocarriers for a wide range of molecular diagnostic probes and therapeutic agents. Both hydrophilic and hydrophobic compounds can be encapsulated into the aqueous inner void of their membrane or within the phospholipid bilayer, respectively.^[1]^ This ability of incorporating molecules with different solubilities, together with their excellent biocompatibility, stability and pharmacokinetics, make liposomes a powerful tool for targeted drug delivery and imaging.^[2]^ The insertion of photosensitizer (PS) molecules into liposome nanocarriers, for example, is an efficient strategy to render them soluble and non‐aggregated, which in turn is crucial to maintain their photophysical properties in photodynamic therapy (PDT) and fluorescence imaging applications.[^3^, ^4^, ^5^, ^6^, ^7^]

In addition to their role as nanocarriers, both artificial and cell‐derived lipid vesicles have been used as closed‐volume nanoreactors and protocell models, to study the structure, function, distribution and binding of proteins in a cell‐mimicking membrane.[^8^, ^9^] These membrane model systems have been investigated alone or in conjunction with supported lipid bilayers (SLBs), allowing the study of other processes such as intermembrane interactions during exo‐ and endocytosis, membrane fusion, and interactions with elements of the cytoskeleton.[^10^, ^11^] In such approaches, the SLB can either interact with the solid support^[12]^ or be tethered to it,^[13]^ while there are strategies (e.g., Langmuir transfer or membrane fusion) to create bilayers with different lipid mixtures in the two leaflets.^[14]^ Several groups have also explored the tethering of vesicles onto SLBs using DNA hybridization^[15]^ or biotin‐streptavidin[^16^, ^17^] recognition elements. In systems displaying multiple copies of the carbohydrate sialic acid (SA), the resulting models have been used to assess important features of the Influenza virus cell infection process, e.g., its multivalent binding and fusion, which show a cooperative dependence on surface density and conformational aspects of receptors within the membrane.[^18^, ^19^]

Another important group of transmembrane receptors that specifically bind sialylated glycans are siglecs (sialic acid‐binding immunoglobulin‐like lectins).[^20^, ^21^] The family of human siglecs is composed of 15 different types, which display diverse biological functions in interactions with pathogens, autoimmune diseases, neurodegeneration, brain disorders and tumoral processes.[^22^, ^23^, ^24^, ^25^, ^26^] Siglec‐10, among others, shows for example immunoreceptor tyrosine‐based inhibitory motifs in the cytoplasmic region, which have a large influence in the evasion of tumors from a potential immune response,[^20^, ^21^] mainly through high expression levels of SA in cancerous tissues.[^27^, ^28^] The use of SA‐containing biomimetic compounds and nanoparticles is therefore an interesting approach to inhibit this siglec‐mediated tumor mechanism of immune scape.^[29]^

**Figure 1:**
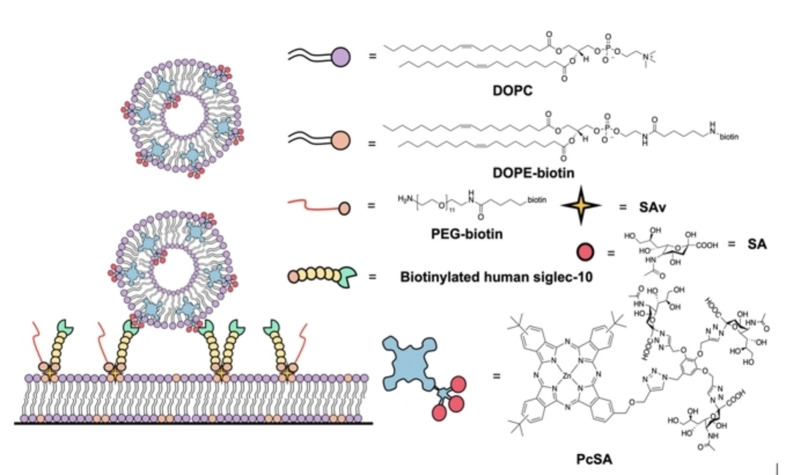
Schematic representation of the binding of DOPC‐Pc SUVs to a siglec‐10‐based SLB, and chemical structures of all components used. Acronyms: DOPC, 1,2‐dioleoyl‐sn‐glycero‐3‐phosphocholine; DOPE‐biotin, 1,2‐dioleoyl‐sn‐glycero‐3‐phosphoethanolamine‐N‐(cap biotinyl); SAv, streptavidin; PEG‐biotin, amino undeca(ethylene glycol)‐biotin; SA, sialic acid.

On those bases, herein we describe how multivalency, a key principle in Nature to control binding and other processes through multiple reversible molecular recognition events,[^30^, ^31^, ^32^, ^33^, ^34^] can be used to tune the interaction of vesicle nanocarriers with human siglec‐10‐displaying SLBs (Figure 1). To this end, 1,2‐dioleoyl‐sn‐glycero‐3‐phosphocholine (DOPC) small unilamellar vesicles (SUVs) have been equipped with a multifunctional (targeting, imaging, and photosensitizing) sialic acid‐modified zinc phthalocyanine derivative (**PcSA**). Phthalocyanines (Pc), chromophores belonging to the family of porphyrinoids, are robust photosensitizers (PS) with near‐infrared optical and photophysical properties that make them ideal for PDT.[^35^, ^36^, ^37^, ^38^] The outstanding properties of liposomes as nanocarriers, loaded with a photoactive dendritic structure that displays SA units for targeting, turn these biohybrids into promising theranostic agents for tumoral and inflammatory processes, which could take advantage of both the phototoxic and fluorescent properties of compound **PcSA**.

## Results and Discussion

### Design of the System

The synthesis, characterization and photodynamic properties of **PcSA** were reported recently.^[39]^ Figure S1 shows data about this compound that complement the present approach to encapsulate it in liposome nanocarriers and to use them as platforms for multivalent targeting of biological receptors, including cell internalization and subcellular localization studies, generation of reactive oxygen species (ROS) in the cells, and phototoxicity experiments against three different superficial tumor cell lines (i.e., A431, HeLa and *S*CC‐13 cells). These studies revealed that by connecting the ZnPc core to a single dendron with three SA units, cell internalization is highly efficient and, once internalized, the photosensitizer accumulates in the lysosomes (Figure S1a–c). Moreover, thanks to its amphiphilic character and the bulky *tert*‐butyl groups, which disrupt aggregation in the lipid bilayer of the lysosome membranes, the compound remains non‐aggregated in such hydrophobic environment, and this maximizes its capacity for ROS generation (Figure S1d) and its photodynamic activity (Figure S1b). From that point, what was missing from the picture is how the photosensitizer could be transported and address the cells. The rationale herein is that the next step implies encapsulation in liposome nanocarriers, with the idea of using them not only as transporters but also as platforms enabling multivalency in the interaction with cellular receptors (e.g., siglecs). Importantly, the strategy of connecting the targeting moieties to the photosensitizer rather than to the liposome lipids allows combining the extracellular targeting of siglecs with a precise subcellular localization and intracellular activation in the lysosomes.

Now, in order to investigate the possible multivalent interactions between human siglec‐10 and **PcSA**, in the present work a DOPC‐based SLB, doped with different mol % of 1,2‐dioleoyl‐sn‐glycero‐3‐phosphoethanolamine‐N‐(cap biotinyl) (DOPE‐biotin), was formed over a substrate in a quartz crystal microbalance with dissipation monitoring (QCM‐D) chamber. The inclusion of DOPE‐biotin was performed with the aim of subsequently binding streptavidin (SAv), owing to the strong affinity of biotin‐SAv that has been well documented in the literature.[^40^, ^41^] The presence of SAv in the SLB allows the incorporation of biotinylated human siglec‐10, again through biotin‐SAv interactions. The fraction of DOPE‐biotin in the SLB is thereby a tuning knob for the siglec density expressed at the SLB surface. The binding of amino undeca(ethylene glycol)‐biotin (PEG‐biotin) to the SAv pockets, in turn, has an important role in the control of the siglec‐10 density in the SLB, and in the suppression of non‐specific interactions between the SUVs and the SLB, as will be explored later. Finally, the multivalent binding between human siglec‐10‐coated SLBs and Pc‐loaded SUVs has been studied through the flow of DOPC‐Pc SUVs over these SLBs, while monitoring changes of the frequency (Δ*f*) and dissipation (Δ*D*) of the QCM‐D sensor. Figure 1 shows a schematic representation of all the components of the system, and the binding of the photosensitizing DOPC‐Pc SUVs to the human siglec‐10‐modified SLB.

### Preparation and Characterization of SUVs with PcSA

DOPC‐Pc SUVs (0.5 mg mL^−1^) of 100 nm in diameter were prepared with different percentages of **PcSA**: 0.1, 0.2, 0.5, 1, 2 and 5 mol %. The SUVs admit higher amounts of encapsulated **PcSA**, but above 5 % the fluorescence and other photodynamic properties of the photosensitizer are quenched due to aggregation (see below), and so it is not worthy to further increase the loading percentage. Initially, **PcSA** was mixed with the lipid amphiphile at the same stage of the vesicle preparation protocol (so‐called direct encapsulation approach, which is described in detail in the Experimental Procedures section, Supporting Information). In brief, the lipid was hydrated using a solution of **PcSA** in PBS (pH 7.4), and vesicles of approx. 100 nm containing different mol % of **PcSA** were obtained. Alternatively, it was hypothesized that **PcSA** could be able to enter into the membrane of previously prepared DOPC‐SUVs, due to its inherent amphiphilic character. To address this possibility, DOPC‐SUVs of 100 nm were also prepared without Pc derivative. Afterwards, they were mixed with the same concentrations of **PcSA** in PBS as in the direct encapsulation experiment, with the aim to achieve insertion and to provide information on the non‐covalent interaction between the Pc compound and preformed SUVs (so‐called insertion approach).

**Figure 2:**
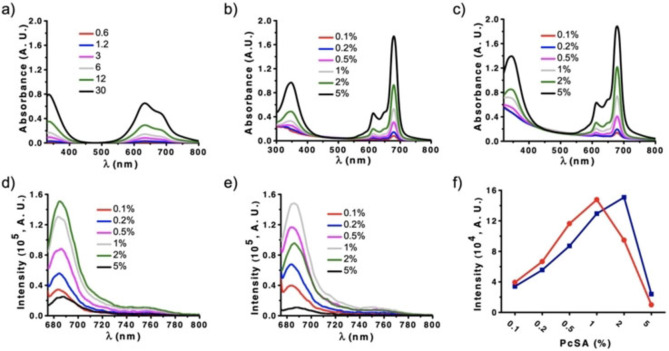
a) Absorption spectra of the **PcSA** in PBS, with concentrations (in μM) equal to those employed for encapsulation in SUVs. b) Absorption spectra of DOPC‐Pc SUVs containing different mol % of **PcSA**, prepared by the direct encapsulation approach. c) Absorption spectra of DOPC‐Pc SUVs containing different mol % of **PcSA**, prepared by the insertion approach. d) Fluorescence spectra of DOPC‐Pc SUVs containing different mol % of **PcSA**, prepared by direct encapsulation approach. e) Fluorescence spectra of DOPC‐Pc SUVs containing different mol % of **PcSA**, prepared by insertion approach. f) Comparison of the fluorescence intensity of DOPC‐Pc SUVs with different mol % of **PcSA**, prepared by direct encapsulation (blue) or insertion (red) methods.

Prior to UV/Vis and fluorescence characterization of the DOPC‐Pc SUVs, control absorption and emission spectra of a solution containing only the DOPC vesicles (without **PcSA**) and of solutions only containing varying concentrations of **PcSA** (without vesicles) were recorded. For the latter, the chosen concentrations ranged from 0.6 μM to 30 μM, matching those used to prepare the Pc‐containing vesicles, to test the aggregation behavior of the **PcSA** derivative under these conditions. Importantly, the UV/Vis spectra obtained at all concentrations showed an absorption maximum at 635 nm (Figure 2a), indicative of H‐type Pc stacking.^[42]^ Quenching of fluorescence across the entire concentration range confirmed the exciton coupling associated with Pc aggregation (Figure S2a). In turn, the absorption and emission spectra of DOPC SUVs (0.5 mg mL^−1^) showed the typical scattering and lack of fluorescence, respectively (Figure S2b and S2c).

In comparison to the individual components of our system, and despite the strong scattering provoked by the vesicles, clear changes could be observed in the absorption and fluorescence spectra of DOPC‐Pc SUVs prepared by direct encapsulation. In particular, the Pc Q‐band was found to be sharp and centered at 679 nm, which indicates a non‐aggregated state of the **PcSA** compound within the vesicle membrane (Figure 2b). In the emission spectra, on the other hand, there was a clear recovery of fluorescence, which correlated well with the increase in mol % of the Pc inside the SUVs (Figure 2d). These results demonstrate that the encapsulation of **PcSA** in DOPC vesicles avoids Pc aggregation in the aqueous medium, suggesting the potential of the vesicles as suitable nanocarriers of this dendritic PS.

For the analysis of DOPC‐Pc SUVs prepared with the insertion approach, similar trends were observed in both the UV/Vis absorption and emission changes, confirming that the addition of **PcSA** to preformed vesicles also contributes to disrupt the Pc stacking (Figure 2c and e). However, interesting insights are obtained when comparing the fluorescence spectral features of both types of Pc‐loaded SUVs. In the direct encapsulation experiments, the intensity of fluorescence was drastically reduced for the highest concentration of **PcSA** (5 mol %, 30 μM). This is tentatively interpreted as a saturation point of DOPC SUVs with the maximum amount of **PcSA** that they can host, above which excited state self‐quenching of the Pc moiety occurs inside the membrane. A fraction of 2 mol % (12 μM) is the optimum concentration of **PcSA** for not affecting the Pc emission properties in the vesicles (Figure 2f, blue curve). The situation is different for the insertion approach, where the maximum of fluorescence recovery was observed at a Pc concentration of 6 μM (1 mol %) (Figure 2f, red curve). The difference in efficiency is tentatively ascribed to the loading of **PcSA** solely into the outer leaflet in case of the insertion approach, while the compound is distributed over both leaflets in case of the direct encapsulation approach.

**Figure 3:**
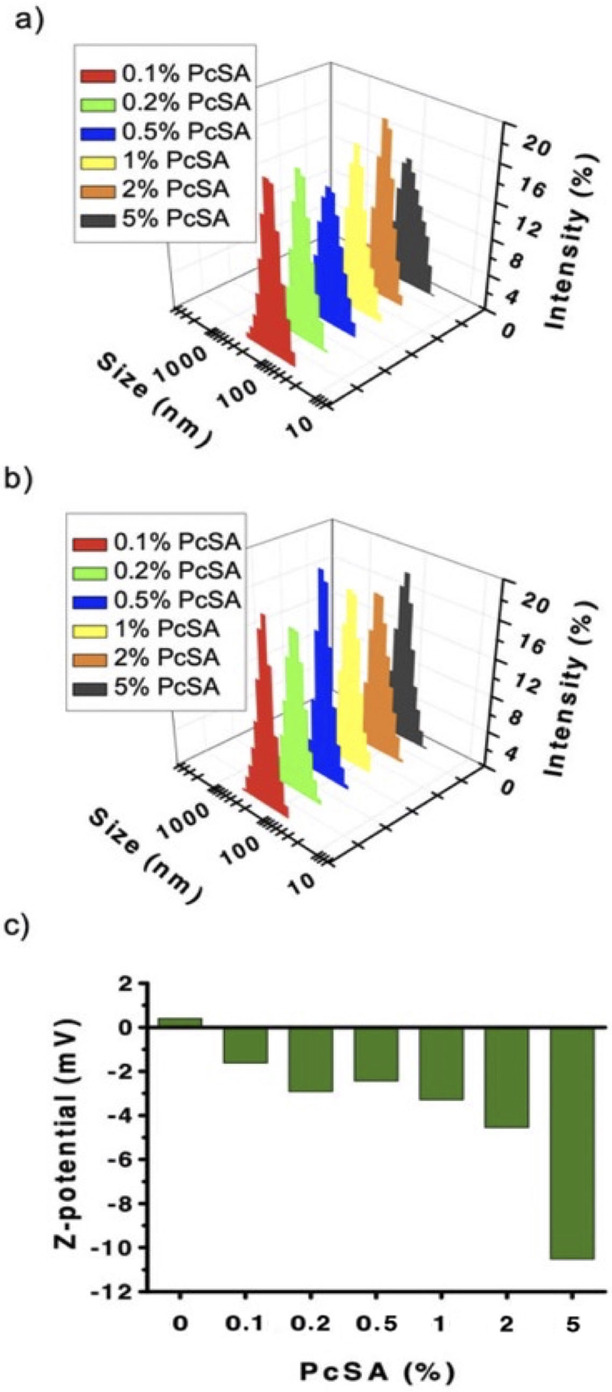
DLS data of DOPC‐Pc SUVs with different mol % of **PcSA**, prepared by a) direct encapsulation or b) insertion experiments. c) Z‐potential values of DOPC‐SUVs and DOPC‐Pc SUVs after the inclusion of **PcSA** in the process of formation of the vesicles with the direct encapsulation approach.

DLS characterization of the prepared DOPC‐Pc SUVs, either by direct encapsulation (Figure 3a) or through insertion in preformed DOPC‐SUVs (Figure 3b), showed well‐defined and monodisperse vesicle sizes with diameters around 100 nm. As a control, the hydrodynamic diameter of **PcSA** alone in PBS, at a low concentration of 5 μM for minimizing the Pc absorption, revealed rather monodisperse assemblies with an average diameter of only 48±11 nm (Figure S3). We note that a peak for the latter assembly size was absent in the DOPC‐Pc SUVs mentioned above, which only showed a peak for the vesicles. Therefore, these results support the successful incorporation of **PcSA** into the vesicles.

The capacity of **PcSA** to aggregate in aqueous buffer is related to its inherent amphiphilic character, and the morphology of such aggregates (irregular spherical assemblies) has been studied previously.^[39]^ Another proof of the incorporation of **PcSA** in the DOPC‐SUVs came from analysis of the Z‐potential of the resulting hybrid vesicles in the encapsulation approach. Because SA is a negatively charged carbohydrate, the incorporation of increasing amounts of **PcSA** should result in increasingly negative Z‐potential values. Figure 3c precisely shows this trend, the Z‐potential values ranging from 0.5 mV in the case of DOPC‐SUVs without Pc to −10.5 mV for DOPC‐Pc SUVs with 5 mol % of Pc. Since the fluorescence properties observed above are indicative of a molecularly dissolved, non‐aggregated Pc, while the sugar moieties appear to be on the vesicles’ surface, according to the increasing negative zeta potential values for vesicles with increasing **PcSA** loadings, the overall interpretation of these results is that the Pc is embedded in the hydrophobic leaflet and the charged polar sugar is in the surrounding aqueous layer (see Figure 1).

### Binding of DOPC‐Pc SUVs to Siglec‐Modified SLBs

**Figure 4:**
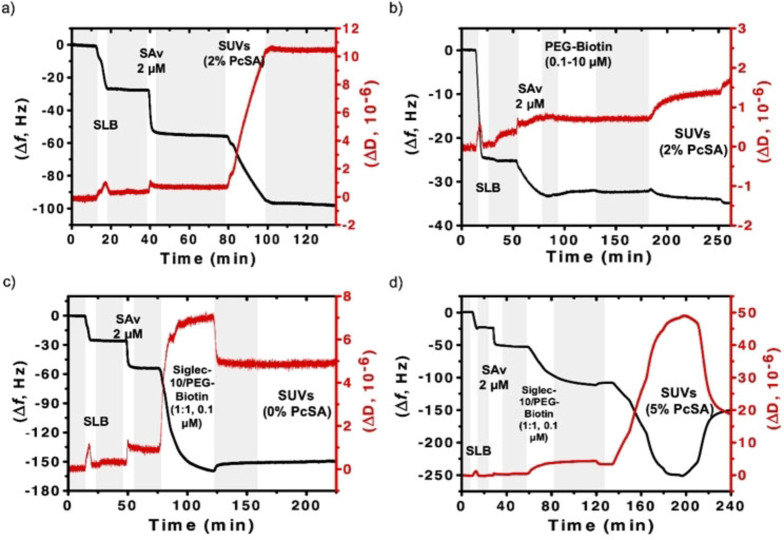
QCM‐D measurements showing frequency shifts (Δ*f*, black) and variations in dissipation (ΔD, red) for: a) formation of a SLB containing 2 % of DOPE‐biotin, adsorption of SAv and binding of DOPC‐Pc SUVs containing 2 % of **PcSA** (dilution 1 : 10). b) Formation of a SLB containing 2 % of DOPE‐biotin, adsorption of SAv and PEG‐biotin, and flow of DOPC‐Pc SUVs containing 2 % of **PcSA** (dilution 1 : 5). c) Formation of a SLB containing 2 % of DOPE‐biotin, adsorption of SAv and a mixture (1 : 1) of PEG‐biotin and biotinylated human siglec‐10, and flow of DOPC‐Pc SUVs containing 0 % of **PcSA** (dilutions 1 : 100–1 : 2). d) Formation of a SLB containing 2 % of DOPE‐biotin, adsorption of SAv and a mixture (1 : 1) of PEG‐biotin and biotinylated human siglec‐10, and binding of DOPC‐Pc SUVs containing 5 % of **PcSA** (dilutions 1 : 100–1 : 2). The 5th overtone was used in all experiments. Grey shades represent PBS flows.

The interaction of DOPC‐Pc SUVs with the SLB platform, functionalized with human siglec‐10, was monitored using QCM‐D. Initially, a DOPC‐SLB was formed, containing 2 % of DOPE‐biotin. This substrate was incubated with SAv (2 μM), which gave a frequency change of Δ*f*=25 Hz, which is typical for an SLB densely packed with SAv.^[43]^ After a wash with PBS, the SLB was incubated with 0.1 μM of biotinylated siglec‐10, revealing a Δ*f*=70 Hz, which confirmed the stable anchoring of siglec proteins to the bilayer (Figure S4a). Prior to further binding studies with DOPC‐Pc SUVs, two control experiments were performed to evaluate the possible existence of non‐specific interactions of the Pc‐loaded vesicles with the SLB in absence of siglec‐10. In the first control, a DOPC‐based SLB without biotin‐DOPE was formed, and SAv (2 μM) and DOPC‐Pc SUVs containing 2 % of **PcSA** were flown over the substrate (Figure S4b). No changes of frequency were detected, indicating that there is no interaction with the vesicles if SAv is not properly attached to the SLB. This is an important result because it indicates that non‐specific interactions are a consequence of the presence of SAv, which in the present design is used to attach the siglecs to the SLB, yet SAv would not be present in the biological environment where these DOPC‐Pc SUVs will be used as nanocarriers. In the second control, the DOPC‐SLB with 2 % of DOPE‐biotin and after adsorption of SAv (2 μM), but in absence of siglec‐10, was incubated with DOPC‐Pc SUVs containing 2 % of **PcSA**. A shift in frequency of Δ*f*>40 Hz was measured in this case, indicating the occurrence of non‐specific binding between DOPC‐Pc SUVs and the highly SAv‐loaded SLB (Figure 4a and S5).

With the aim to avoid the above non‐specific interaction, two different strategies were considered for tuning the SAv/siglec‐10 density on the SLB. The first one consisted of loading the SLB with a lower mol % of DOPE‐biotin. For example, using 0.2 % of DOPE‐biotin gave a Δ*f*=7 Hz after the incubation with SAv, and a Δ*f*=20 Hz after the binding of biotinylated siglec‐10 (Figure S6a), both corresponding to roughly 30 % of the frequencies obtained on a 2 % DOPE‐biotin SLB. On the other hand, a dilution with PEG‐biotin, achieved by using a 1 : 1 mixture with biotinylated siglec‐10 to achieve the siglec‐presenting SLB, gave about half of the frequency in comparison with the use of only biotinylated siglec‐10 (Figure S6b). This last approach was proposed to suppress the non‐specific binding between the DOPC‐Pc SUVs and the SAv‐modified SLBs. To verify such hypothesis, 10 μM of biotin‐PEG was flown over a DOPC‐based SLB doped with 0.2 % of DOPE‐biotin and pre‐incubated with 2 μM of SAv (Figure 4b). It is noteworthy that the binding of PEG‐biotin cannot be registered due to the small molecular weight of this compound, which also explains why diluting siglec‐10 with PEG‐biotin in a 1 : 1 ratio gives half of the signal (shown above). Thereafter, the modified SLB was incubated with DOPC‐Pc SUVs containing 2 % of **PcSA**, and only a small shift of Δ*f*=2 Hz was observed (Figure 4b). This last result indicates that biotin‐PEG helps fine‐tuning the density of siglec‐10 moieties displayed on the SLB platform and, at the same time, suppresses the non‐specific interaction of the DOPC‐Pc SUVs with the siglec‐10‐displaying SLB.

The next step was to study the possible multivalent interaction of DOPC‐Pc SUVs presenting different percentages of **PcSA** (0, 1, 3 and 5 %, compared in Figures S7–S10, respectively) with siglec‐10 displayed on SLBs, which in turn were modified with different mol % of DOPE‐biotin (0.5, 1 and 2 %, shown in panels a, b and c of Figures S7–S10). In all cases, the SLBs were incubated with 2 μM of SAv and a mixture (1 : 1) of PEG‐biotin and biotinylated siglec‐10 (0.1 μM). Figure 4c shows the control of flow of DOPC SUVs containing 0 % of **PcSA** over a DOPC‐based SLB modified with 2 % of DOPE‐biotin, followed by the addition of SAv and the mixture (1 : 1) of PEG‐biotin and biotinylated siglec‐10. The lack of response when SUVs without **PcSA** were added proved the need of displaying SA targeting moieties on the vesicles for efficient binding. Figure 4d, in turn, shows the same experiment but using DOPC‐Pc SUVs containing 5 % of **PcSA**, and a clear Δ*f* was observed up to a value of 150 Hz. The experiments with intermediate concentrations of **PcSA** (1 and 3 %) are presented in Figures S8c and S9c, and gave rise to Δ*f* values of 2 and 100 Hz, respectively. Such frequency data, and the observed dissipation values, are indicative of the binding of intact vesicles to the SLB^[41]^ and the achievement of dense vesicle packing. Overall, the data confirm the binding of Pc‐loaded vesicles to siglec‐10‐presenting SLBs by specific SA‐siglec interactions. It is also noteworthy that experiments performed at 0.5 % of biotin‐DOPE indicated very low frequency changes, even at 5 % of **PcSA** in the vesicles, confirming excellent antifouling properties. At increased mol % of **PcSA** and increased biotin‐DOPE, initial (and large) frequency decreases (indicating adsorption) were occasionally followed by reversing positive frequency changes, which are tentatively attributed to vesicle rupture occurring at high affinity and high vesicle packing density, but this was not investigated further.

**Figure 5:**
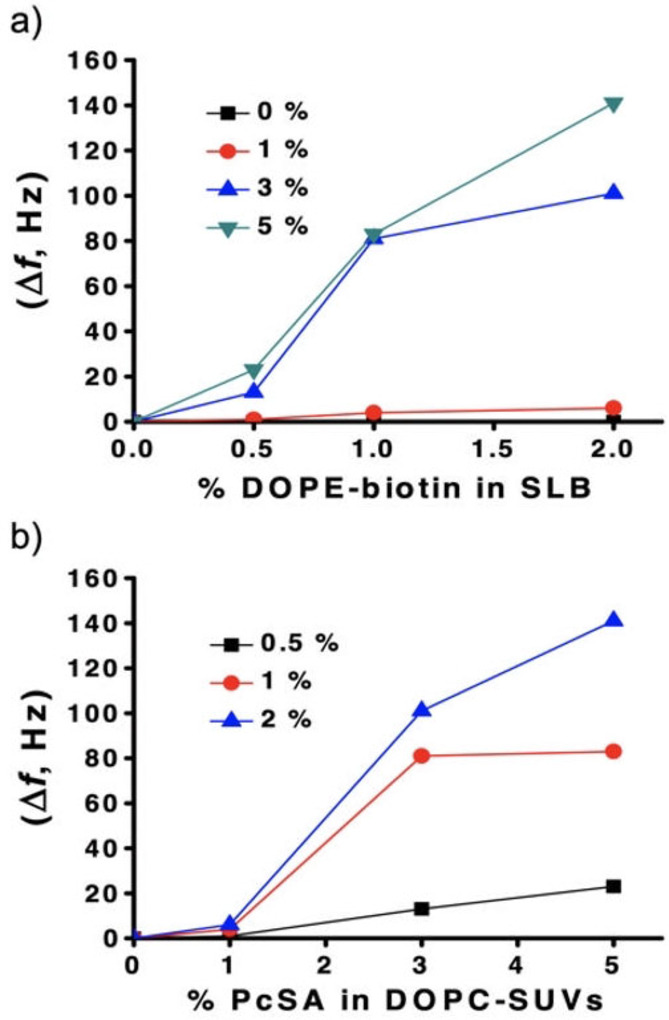
a) Plot of Δ*f* vs the mol % of DOPE‐biotin in the SLB, with different mol % of **PcSA** in DOPC‐SUVs. b) Plot of Δ*f* (absolute values) vs the mol % of **PcSA** in DOPC‐SUVs, with different % of DOPE‐biotin at the SLB. The estimated standard deviation of Δ*f* holds an upper limit of 20 %.

Plotting the obtained Δ*f* values versus the mol % of DOPE‐biotin displayed on the SLB shows no interaction when the mol % of **PcSA** in the DOPC‐SUVs is equal to 0, and a clear dependence of SUV binding on the siglec‐10 density, with a rather steep transition between 1 % and 3 % of **PcSA** (Figure 5a). Likewise, the same series indicated a transition between hardly any binding at 0.5 % of biotin in the SLB vs more efficient binding at 1 and 2 mol % of biotin‐DOPE. If the same Δ*f* data are plotted as function of the **PcSA** mol % displayed on the DOPC‐SUVs, both trends are visible as well (Figure 5b).

Both dependencies appear to be non‐linear, which is a strong indication that the SA‐siglec‐10 interactions in the present system are multivalent and specific. We attribute the observed trends to superselective binding of the vesicles to the SLBs.^[44]^ The sigmoidal trends constitute a hallmark of weakly multivalent binding^[31]^ and signify the occurrence of threshold densities, in both the vesicle and SLB, above which binding becomes efficient. This type of multivalent binding is reminiscent of the binding of influenza virus particles to cell surfaces, and has been experimentally verified and quantified.^[45]^ The rather similar type of interaction used here (sialoglycan‐protein interactions), the similar particle size (about 100 nm) and the similar threshold densities of the transitions, hint at the SUV‐siglec system to have an individual binding affinity (in the mM range) and binding valency similar to the virus binding case (but further work would be needed to explore this in a more quantitative fashion).

## Conclusion

In this work, we have tailored the multivalent association of human siglec‐10 displayed on SLBs with DOPC‐Pc SUVs presenting photoactive SA dendrons at the outer surface of their membrane. The binding was evidenced by strong frequency changes measured with QCM‐D. The binding is strongly and non‐linearly dependent on the siglec‐10 density displayed on the SLB surface and on the density of **PcSA** presented at the membrane of the DOPC‐SUVs. In fact, threshold densities were observed, which indicate the occurrence of weakly multivalent interactions and constitute the hallmark of superselective binding.^[44]^ Most importantly, the occurrence of the threshold densities provides a potential for selective targeting: at the proper Pc fraction, the vesicles have a preference for an SLB (or a cell membrane) with a sufficiently high receptor concentration, hence this mechanism conceptually promises selectivity for binding cells with over‐expressed receptor densities.[^33^, ^34^] The incorporation of **PcSA** into the membrane of DOPC SUVs also allows the recovery of its photophysical properties, a fact that is normally crucial for the fluorescence and ROS generation activity of PS in biological media. Indeed, **PcSA** presents excellent PDT activity against different superficial cancer cell lines, as previously shown.^[39]^ The present study thus highlights an avenue to the use of these highly biocompatible DOPC‐Pc SUVs in biomedical applications, as powerful agents for specific targeting and delivery of PS into tumoral and inflammatory processes where members of the siglec family are involved.

## Conflict of interest

The authors declare no conflicts of interest.

1

## Supporting information

As a service to our authors and readers, this journal provides supporting information supplied by the authors. Such materials are peer reviewed and may be re‐organized for online delivery, but are not copy‐edited or typeset. Technical support issues arising from supporting information (other than missing files) should be addressed to the authors.

Supporting Information

## Data Availability

The data that support the findings of this study are available in the Supporting Information of this article.
