# Supplementary material for: Tailored Multivalent Targeting of Siglecs with Photosensitizing Liposome Nanocarriers
Source: Angew Chem Int Ed Engl. 2022 Jun 21;61(31):e202206900. doi: 10.1002/anie.202206900 (PMC9401027; doi:10.1002/anie.202206900)
Supplement: Supplementary file 1 — Supporting Information [file ANIE-61-0-s001.pdf]

Angewandte Chemie
Eine Zeitschrift der Gesellschaft Deutscher Chemiker

Supporting Information

Tailored Multivalent Targeting of Siglecs with Photosensitizing Liposome Nanocarriers

V. Almeida-Marrero, F. Bethlehem, S. Longo, M. C. Bertolino, T. Torres, J. Huskens*, A. de la Escosura**

Supporting Information

Table of Contents

- **Experimental Procedures**…………... 2
- **Figure S1**………………………………. 3
- **Figure S2**………………………………. 4
- **Figure S3**………………………………. 4
- **Figure S4**………………………………. 5
- **Figure S5**………………………………. 5
- **Figure S6**………………………………. 5
- **Figure S7**………………………………. 6
- **Figure S8**………………………………. 7
- **Figure S9**………………………………. 7
- **Figure S10**………………………………8

Experimental Procedures

Materials

Chemicals were purchased from Sigma-Aldrich, otherwise indicated. 1,2-Dioleoyl-sn-glycero-3-phosphocholine (DOPC), and 1,2-dioleoyl-sn-glycero-3-phosphoethanolamine-N-(cap biotinyl), sodium salt (DOPE-biotin) were purchased from Avanti Polar Lipids. Ultrapure water (Milli-Q) obtained by ultrafiltration was used in all the experiments. PBS (0.01 M sodium dihydrogen phosphate and 0.15 M sodium chloride, pH 7.4) was prepared dissolving commercial PBS tablets in ultrapure water. Streptavidin was purchased from ThermoFisher and dissolved in PBS. Biotinylated human siglec-10 was purchased from Acro Biosystems and dissolved in PBS to reach a 0.1 µM solution. Amino undecaethylene glycol-biotin (PEG-biotin) was purchased from ThermoFisher.

Preparation of Small Unilamellar Vesicles (SUVs)

Batches of 1 mL of 0.5 mg/mL of DOPC SUVs in PBS pH= 7.4, with/without different molar percentages of **PcSA** (0.1, 0.2, 0.5, 1, 2 and 5%), were prepared using a stock solution of 25 mg/mL of DOPC in chloroform (with 0-2 mol% DOPE-biotin in case of SUVs for SLB formation), and a stock solution of 1.44 mg/mL of **PcSA** in PBS. After adding the required amount of DOPC in chloroform into a glass vial, a gentle nitrogen flux was used to evaporate the solvent, forming a lipidic film on the vial walls. The residual solvent was removed placing the vials into a desiccator for 1 h. 1 mL of Milli-Q water (in case of SUVs for SLB formation) or PBS (for the binding of SUVs onto SLBs; different ratios of PBS and **PcSA** in PBS were used to obtain the desired %) was added. After performing 10 freeze-pump-thaw cycles, the vesicles were extruded through a 100 nm polycarbonate membrane (11 times; Whatman) and were stored in the fridge for two weeks.

Insertion Approach

DOPC-SUVs (0.5 mg/mL) in PBS were mixed with different concentrations of **PcSA** (0.6, 1.2, 3, 6, 12 and 30 µM) adjusting the volume to 1 mL of PBS pH= 7.4, being the same concentrations used for the formation of DOPC-SUVs containing different mol% of **PcSA** and were incubated for 4 h at room temperature under stirring. Vesicles were stored in the fridge and used within two weeks.

Particle Size Analysis

Particle size was recorded using DLS of 1 mL of DOPC-SUVs and DOPC-Pc SUVs (0.5 mg/mL) containing 0.1, 0.2, 0.5, 1, 2 and 5% of **PcSA** in PBS using a Nanotrac Wave particle size analyzer. The data are expressed as the average of three measurements, for a time of 120 s at 22°C. In the insertion experiment, the particle size of the resulting vesicles was measured using the same parameters mentioned before.

Spectroscopic Experiments

UV-Vis spectra were recorded using a Perkin Elmer Lambda 850 spectrophotometer. 1 mL of every sample of vesicles were used to record the data, in a Hellma 108-QS 1000 µL Semi-Micro Absorption Cell, 10 mm Light Path. Fluorescence spectra of the previous mentioned vesicles were recorded using a Perkin Elmer FL 6500 fluorescence spectrometer, using Hellma 105.250-QS 100 µL Ultra-Micro Fluorescence Cell with 100 µL of every sample and irradiating at 665 nm.

Z-potential Measurements

Z-potential values were obtained using a Malvern Instruments Zetasizer Nano ZS and a folded capillary Zeta Cell from Malvern Panalytical. Samples were obtained diluting 800 µl of vesicles (0.5 mg/mL) 20 times with ultra-purified water, and Z-potential values were expressed as an average of 3 measurements at 25°C of 800 µL of every sample.

QCM-D Measurements

For the fabrication of SLBs, vesicles (SUVs) were diluted to a concentration of 0.1 mg/ml in PBS directly before use. SLBs were obtained by flowing this solution on a cleaned and activated SiO_2 surface, after obtaining a stable baseline. The quality of the SLBs was monitored in situ by QCM-D (where high quality SLBs are defined by $\Delta f = -24 \pm 1$ Hz and $\Delta D < 0.5\times10^{-6}$). QCM-D measurements were performed with a Qsense Analyser from Biolin Scientific, and SiO_2-coated sensors (QSX303, Biolin Scientific) were used throughout this work. Measurements were done at 22 ºC and operated with four parallel flow chambers, using two Ismatec peristaltic pumps with a flow rate of 20 µl/min. For every measurement, the fifth overtone was used for the normalized frequency ($\Delta f5$) and dissipation ($\Delta D5$). In a typical experiment, SLBs were formed on previously cleaned and activated SiO_2 sensors. Afterwards, solutions of SAv, of siglec-10 or siglec-10/PEG (1:1) mixtures, and then DOPC-SUVs or DOPC-Pc SUVs were added until a stable plateau was reached. Each step was followed by rinsing with PBS. The SLB and SAv steps were found reproducible within a standard deviation of 5% over multiple series. The siglec steps were found reproducible within a standard deviation of below 20%. For practical reasons, this % deviation could not be determined for the vesicle steps but based on previous experience,[41,43,45] we can assume the upper limit of the siglec (20% standard deviation) to hold also in this case.

SUPPORTING INFORMATION

Supporting Figures

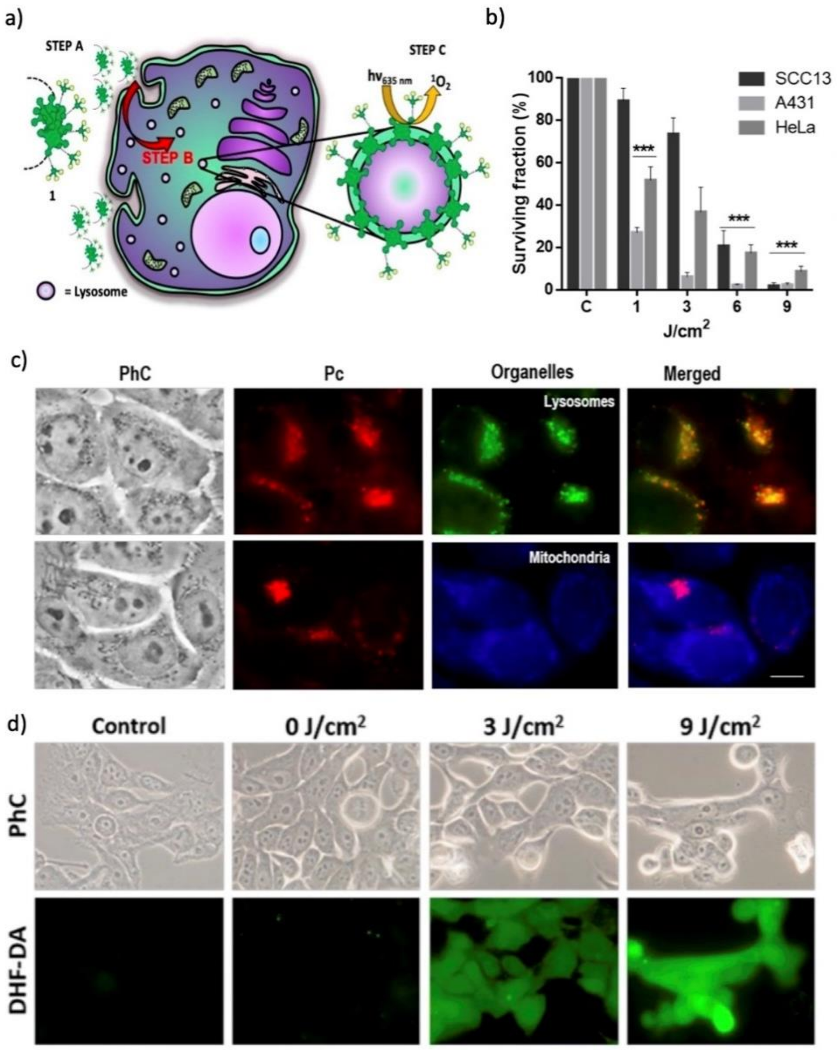

Figure S1. a) Schematic cartoon representing the mechanism of internalization and subcellular behavior of the **PcSA** photosensitizer, according to previous PDT studies by de la Escosura, Torres and coworkers,[1] which involve the following steps. STEP A: **PcSA** is aggregated outside the cell. STEP B: The incorporation into the lysosomes provokes the disruption of **PcSA** aggregation. STEP C: On-site activation of the photodynamic properties of **PcSA**. b) Graphic showing the survival of A431, HeLa and SCC-13 cells after 5 h of incubation of **PcSA** at a concentration of 0.5 µM and after different light doses. c) Subcellular localization of **PcSA** (10 µM) after 18 h of incubation in SCC-13 cells; PhC: phase contrast. Blue fluorescence is from mitochondria (after irradiation with UVA exciting lamp of 360-370 nm and the presence of MitoTracker), green fluorescence is from lysosomes (after irradiation with a blue exciting lamp of 450-490 nm and the presence of LysoTracker), and red fluorescence is from **PcSA** (after irradiation with green exciting light of 545 nm). Scale bar: 10 µm. A similar behavior was observed with the two other cell lines. d) ROS production within the cells, detected by DHF-DA fluorescent probe after PDT with **PcSA**, upon irradiation with different red light doses. The first column is a control without **PcSA**, the second column are the cells in the dark as a second control, and the third and fourth columns are the cells irradiated with different light doses. By fluorescence microscopy (λ_{ex} = 436 nm), it can be detected a fluorescence signal that indicates the production of ROS. PhC: phase contrast. Figure adapted with permission from ref. 1 (39 in the main text). Copyright 2021 Wiley.

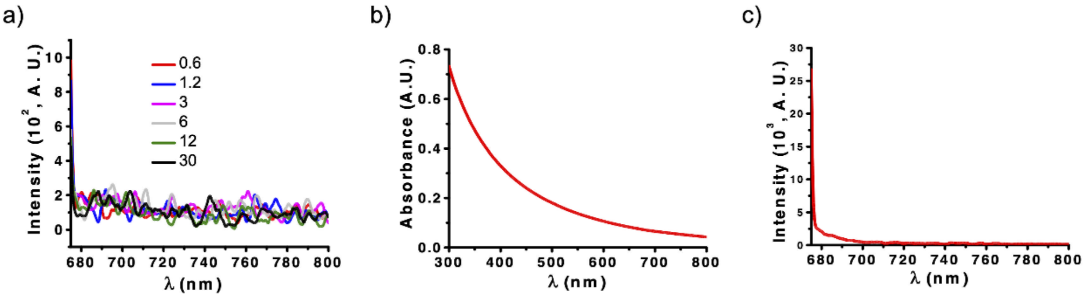

Figure S2. a) Fluorescence emission of **PcSA** in PBS, with concentrations (μM) equal to those employed for encapsulation in SUVs (irradiation at 665 nm). b) Scattering of DOPC SUVs (0.5 mg/mL) in PBS. c) Fluorescence spectra of DOPC SUVs, same conditions (irradiation at 665 nm).

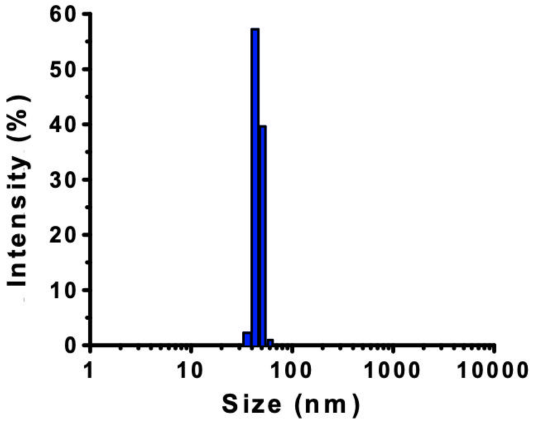

Figure S3. DLS size distribution of **PcSA** (5 μM) in PBS.

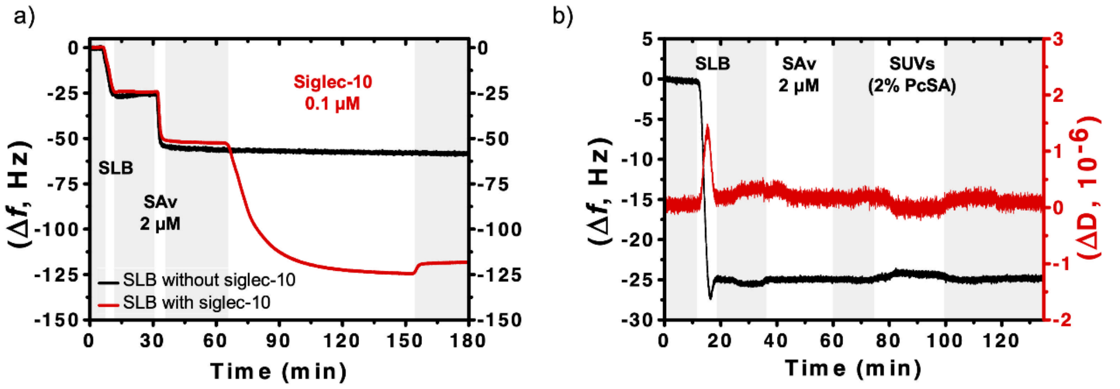

Figure S4. QCM-D measurements showing stepwise: a) Comparison of the frequency shift (Δ*f*) after the addition or not of biotinylated siglec-10 over a DOPC-based SLB displaying 2 % of DOPE-biotin. The frequency shift (Δ*f*) of the SLB displaying siglec-10 is represented in red, while the one corresponding to the SLB not displaying siglec-10 is represented in black. The 5th overtone was used in all the experiments. Grey shades represent PBS addition. b) Formation of a DOPC-based SLB, flow of SAv and DOPC-Pc SUVs containing 2 % of **PcSA** (dilution 1:100 - 1:2), with no interactions observed. The frequency shift (Δ*f*) is represented in black and the variation of dissipation is represented in red (ΔD).

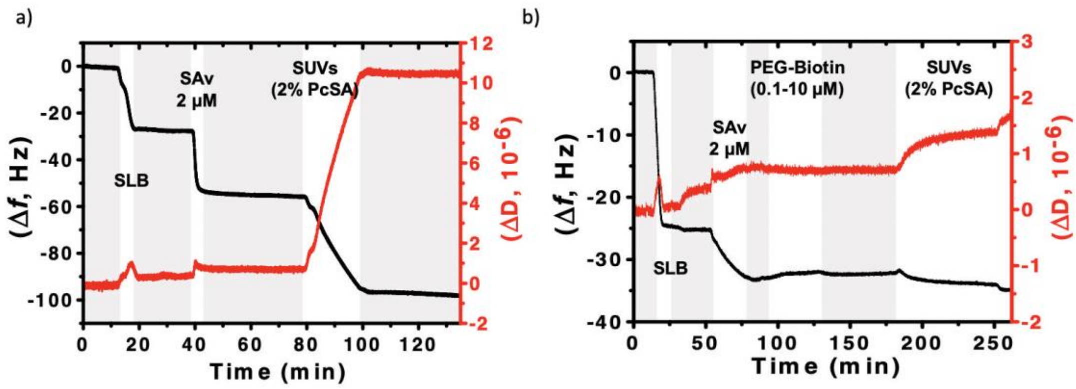

Figure S5. QCM-D measurements showing: a) formation of a SLB containing 2% of DOPE-biotin, adsorption of SAv and binding of DOPC-Pc SUVs containing 2 % of **PcSA** (dilution 1:10). b) Formation of a SLB containing 2% of DOPE-biotin, adsorption of SAv and PEG-biotin, and flow of DOPC-Pc SUVs containing 2 % of **PcSA** (dilution 1:5). The 5th overtone was used in all the experiments. The frequency shift (Δ*f*) is represented in black and the variation of dissipation is represented in red (ΔD). Grey shades represent PBS addition.

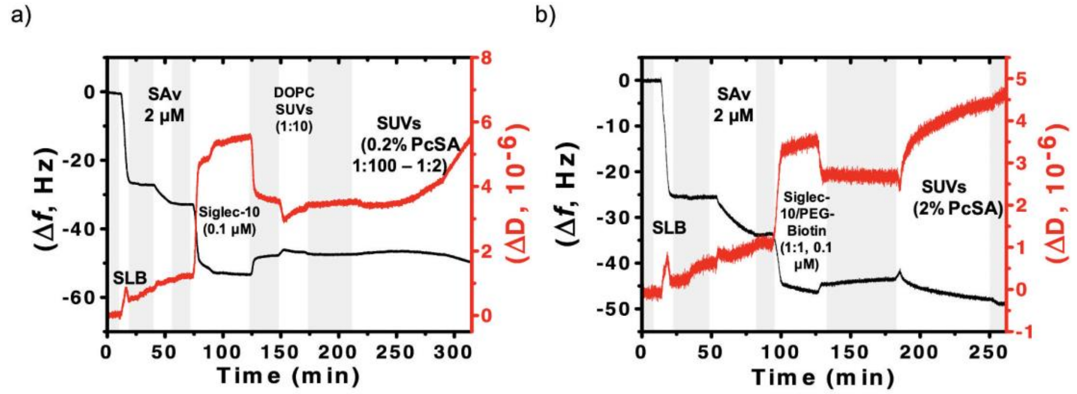

Figure S6. QCM-D measurements showing: a) Formation of DOPC SLB (with 0.2% of DOPE-biotin), followed by adsorption of SAv and biotinylated siglec-10, followed by flowing solutions with DOPC SUVs (without 1) and various dilutions of SUVs containing 0.2% of **PcSA**, both of which did not show any appreciable adsorption. b) Formation of DOPC SLB (with 0.2% of DOPE-biotin), followed by adsorption of SAv and siglec-10 (from a 1:1 mixture of PEG-biotin and biotinylated siglec-10), followed by flowing a solution with SUVs containing 2% **PcSA** (dilution 1:5) showing only minor adsorption. The 5th overtone was used in all the experiments. The frequency shift (Δ*f*) is represented in black and the variation of dissipation is represented in red (ΔD). Grey shades represent PBS addition.

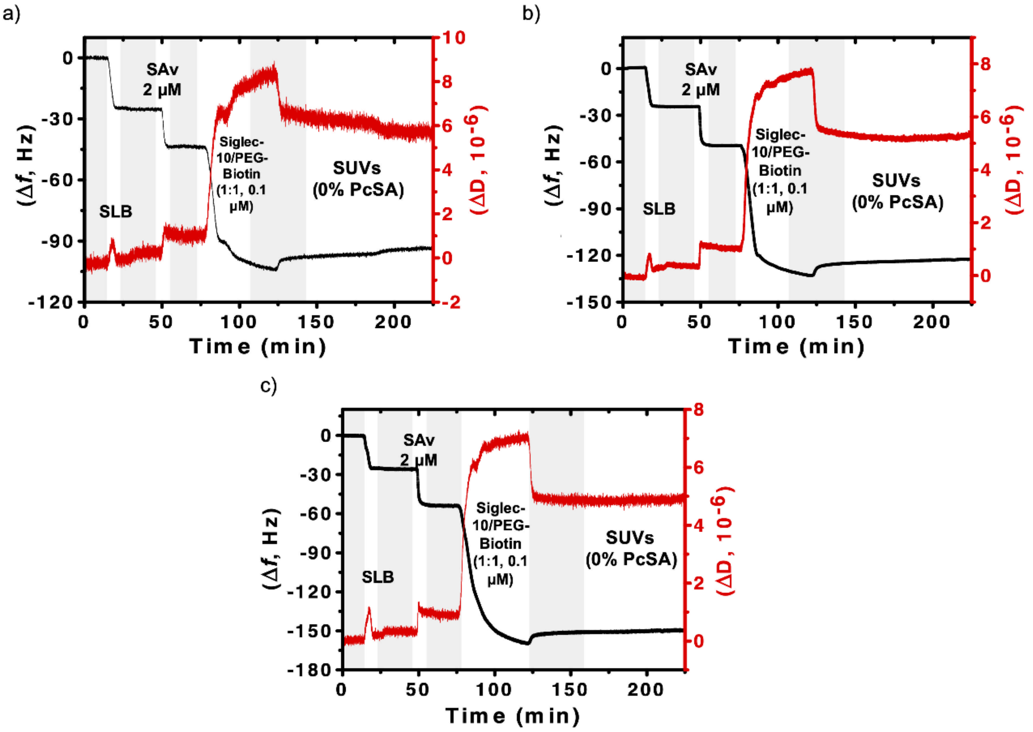

Figure S7. QCM-D measurements showing stepwise: Formation of a SLB, adsorption of SAv and a mixture (1:1) of PEG-biotin and biotinylated human siglec-10, and no binding of DOPC-Pc SUVs containing 0 % of **PcSA** (dilution 1:100 – 1:2), with a) SLB doped with 0.5 % of DOPE-biotin, b) SLB doped with 1 % of DOPE-biotin and c) SLB doped with 2 % of DOPE-biotin. The 5th overtone was used in all the experiments. The frequency shift (Δ*f*) is represented in black and the variation of dissipation is represented in red (ΔD). Grey shades represent PBS addition.

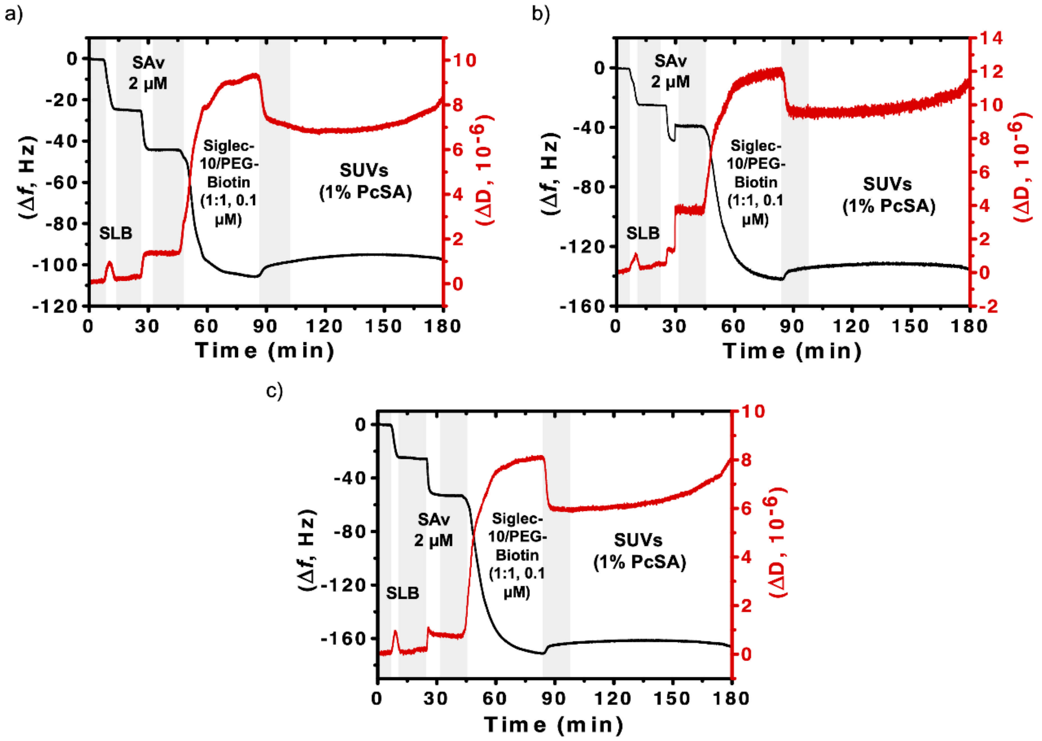

Figure S8. QCM-D measurements showing stepwise: Formation of a SLB, adsorption of SAv and a mixture (1:1) of PEG-biotin and biotinylated human siglec-10, and flow of DOPC-Pc SUVs containing 1 % of **PcSA** (dilution 1:100 – 1:2), with a) SLB doped with 0.5 % of DOPE-biotin, b) SLB doped with 1 % of DOPE-biotin and c) SLB doped with 2 % of DOPE-biotin. The 5th overtone was used in all the experiments. The frequency shift (Δ*f*) is represented in black and the variation of dissipation is represented in red (ΔD). Grey shades represent PBS addition.

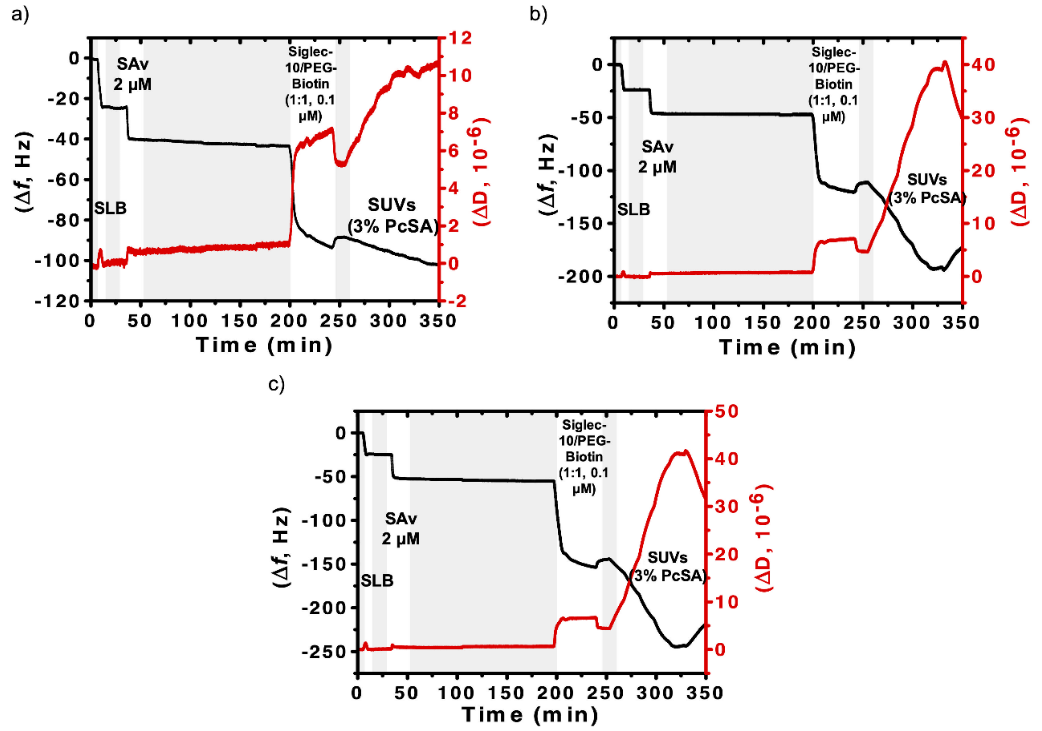

Figure S9. QCM-D measurements showing stepwise: Formation of a SLB, adsorption of SAv and a mixture (1:1) of PEG-biotin and biotinylated human siglec-10, and flow of DOPC-Pc SUVs containing 3 % of **PcSA** (dilution 1:100 – 1:2), with a) SLB doped with 0.5 % of DOPE-biotin, b) SLB doped with 1 % of DOPE-biotin and c) SLB doped with 2 % of DOPE-biotin. The 5th overtone was used in all the experiments. The frequency shift (Δ*f*) is represented in black and the variation of dissipation is represented in red (ΔD). Grey shades represent PBS addition.

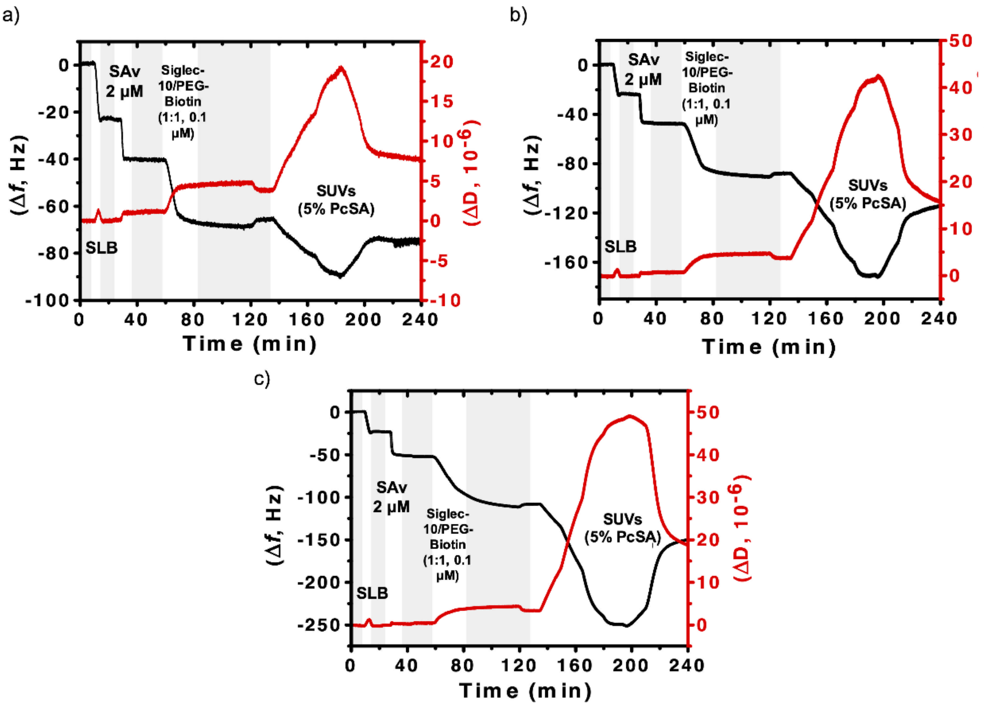

Figure S10. QCM-D measurements showing stepwise: Formation of a SLB, adsorption of SAv and a mixture (1:1) of PEG-biotin and biotinylated human siglec-10, and flow of DOPC-Pc SUVs containing 5 % of **PcSA** (dilution 1:100 – 1:2), with a) SLB doped with 0.5 % of DOPE-biotin, b) SLB doped with 1 % of DOPE-biotin and c) SLB doped with 2 % of DOPE-biotin. The 5th overtone was used in all the experiments. The frequency shift (Δ*f*) is represented in black and the variation of dissipation is represented in red (ΔD). Grey shades represent PBS addition.

References

[1] V. Almeida-Marrero, M. Mascaraque, M. J. Vicente-Arana, A. Juarranz, T. Torres, A. de la Escosura, *Chem. Eur. J.* **2021**, *27*, 9634−9642.
